# Flower Strips in Wheat Intercropping System: Effect on Pollinator Abundance and Diversity in Belgium

**DOI:** 10.3390/insects9030114

**Published:** 2018-09-04

**Authors:** Clara Amy, Grégoire Noël, Séverin Hatt, Roel Uyttenbroeck, Frank Van de Meutter, David Genoud, Frédéric Francis

**Affiliations:** 1 Functional and Evolutionary Entomology, Department of Agronomy, Biology and Chemistry, Gembloux Agro-Bio Tech, University of Liege, Passage des Déportés 2, 5030 Gembloux, Belgium; gregoire.noel@uliege.be (G.N.); severin.hatt@uliege.be (S.H.); frederic.francis@uliege.be (F.F.); 2 TERRA—AgricultureIsLife, Gembloux Agro-Bio Tech, University of Liege, Passage des Déportés 2, 5030 Gembloux, Belgium; roel_uyttenbroeck@hotmail.com; 3 Biodiversity and Landscape, Department of Biosystems Engineering, Gembloux Agro-Bio Tech, University of Liege, Passage des Déportés 2, 5030 Gembloux, Belgium; 4 The Research Institute for Nature & Forest (INBO), Herman Teirlinck building, Venue du Port, 1000 Brussels, Belgium; frank.vandemeutter@kuleuven.be; 5 Diagnostic, Gestion, Expertise (DGE), 10 rue du Président Fallières, 11000 Carcassonne, France; dge-davidgenoud@orange.fr

**Keywords:** sustainable agriculture, ecosystem services, Apoideae, Syrphidae, *Dimorphoteca pluvialis*, *Camelina sativa*, *Coriandrum sativum*, *Fagopyrum esculentum*, *Andrena nitidiuscula*

## Abstract

The decline of pollinators in agricultural areas has been observed for some decades, this being partly due to landscape simplification in intensive agrosystems. Diversifying agricultural landscapes by sowing flower strips within fields could reduce these adverse effects on biodiversity. In this context, the study presented here aimed at assessing and comparing the abundance and diversity of bees (Hymenoptera: Anthophila) and hoverflies (Diptera: Syrphidae) found and visiting flowers in three types of flower strips in Belgium: (i) a mixture of 11 wild flowers, (ii) a monofloral strip of *Dimorphoteca pluvialis* (Asteraceae) and (iii) a monofloral strip of *Camelina sativa* (Brassicaceae), where the last two are considered to be intercrops since they are valuable on the market, all sown within a field of winter wheat (*Triticum aestivum* L.). Pollinators were captured with pan traps and by netting in standardised transects from May to July 2017. One-thousand one-hundred and eighty-four individuals belonging to 43 bee species and 18 hoverfly species were collected. Significant differences in hoverfly diversity were found between the different flower strips. The multifloral treatment supported a greater diversity of syrphid species. Various pollinator species visited the different flowers composing the mixture and also *D. pluvialis*. The pollinator community proved to be predominantly generalist, with the exception of an oligolectic species in Belgium, *Andrena nitidiuscula*. Moreover, the three tested flower strips were effective in attracting hoverflies, among them natural enemies of insect pests. This study opens new perspectives in the design of intercropping systems with flower strips towards the design of sustainable agro-ecosystems. Improving economic profitability of sowing flower strips could encourage farmers to diversify their agricultural systems and foster conservation biology strategies.

## 1. Introduction

With approximately 20,000 species worldwide and more than 2000 species in Europe, bees (Hymenoptera: Anthophila) are among the most speciose pollinator groups in temperate agriculture landscapes [1]. Their long-time coevolution with flowering plants has provided them with both morphological (e.g., scopa, pollen baskets) and behavioural (e.g., lectism, sociality) traits, suitable for plant pollination [2,3]. Non-bee insects, among other hoverflies (Diptera: Syrphidae), are also important for pollinating plants because they are responsible for 25–50% of the total number of visits to flowers [4,5] and contribute significantly to pollination [6,7]. Pollination is an essential ecosystem service because 70% of the diversity of plants cultivated globally and up to 84% of plants cultivated in Europe depend on it [8]. Its economic value has been estimated at 153–285 billion Euros a year [9]. In Belgium, the contribution of insect pollinators to plant production for human food (i.e., mainly fruits and vegetables) was estimated at about 250 million Euros in 2010 [10].

For some 50 years now, pollinator diversity and abundance have been declining at a large scale [11,12]. Important drivers responsible for this decline are the simplification of landscapes and fragmentation of habitats caused by urbanisation processes and agricultural intensification [13]. Indeed, along with the modernisation of agriculture, parcel size has dramatically increased on 40% of the European landscape [14] due to the suppression of semi-natural habitats (i.e., hedges, groves, fallows).

The loss of pollinators from agricultural landscapes threatens the service of pollination [15]. In fact, pollinator decline could negatively impact pollinator-dependent crop yields (e.g., orchards, cultivation of vegetables), creating a negative economic impact [16]. This depletion could have severe implications for producers and consumer welfare [16]. Current pollinator decline may also lead to deficiency of essential minerals and vitamins for the human diet provided by pollinator-mediated crops [17]. Moreover, wild plants could suffer from a dearth of pollination and such effects may cascade further through the food web [18]. These threats could have detrimental effects on agro-ecosystems, human food supply and well-being [16].

In this context, Agri-Environmental and Climate Measures (AECM) have been proposed to farmers in Europe to ‘reduce environmental risks associated with modern farming on the one hand and preserve nature and cultivated landscapes on the other hand’ [19]. Farmers can adopt AECM on a voluntary basis and receive monetary compensation in return for potential losses of income. In Wallonia (Belgium), 11 measures are available to farmers who commit themselves for at least five years [20]. Some of these measures aim at supporting pollinators, such as wildflower strips. Flowering strips are recognised to support insect populations in general [21] and pollinators particularly [22,23], yet their effect depends strongly on the floral composition of the sown mixtures [24]. Previous studies explored how pollinator communities are affected by the species diversity of flower mixtures [25], by the functional diversity of flower mixtures [26] and by specific plant species that are known to be attractive to pollinators [27]. Additionally, spatial diversification of agroecosystems is increasingly considered to improve the sustainability of agriculture [28]. Within fields, intercropping (i.e., the cultivation of at least two crops simultaneously) can reduce the requirement for fertilisers [29] and the risks of infestations by insect pests [30] and diseases [31]. Considering flowering crops in intercropping could moreover benefit pollinators.

The first objective of this study is to estimate the biodiversity of pollinator communities (Hymenoptera: Anthophila and Diptera: Syrphidae) on several flower strips sown in wheat (*Triticum aestivum* (L., 1753)) crops. The second objective is to compare three modalities of flower strips regarding their effect on pollinator abundance and diversity: a multifloral mixture of wildflower species and two oilseed monofloral strips of *Camelina sativa* (Crantz, 1753) (Brassicaceae) and *Dimorphoteca pluvialis* (Moench, 1794) (Asteraceae) are considered intercrops since they are valuable on the market [32,33]. Whereas sowing mixtures of wildflowers can be subsidised through the AECM, the latter two options would offer opportunities of income diversification to farmers [34,35]. By focusing on bees and hoverflies more particularly, the third objective of the present study is to explore how these two groups of pollinators interact with the different floral species within the flower strips.

## 2. Materials and Methods

### 2.1. Experimental Setup

**Figure 1:**
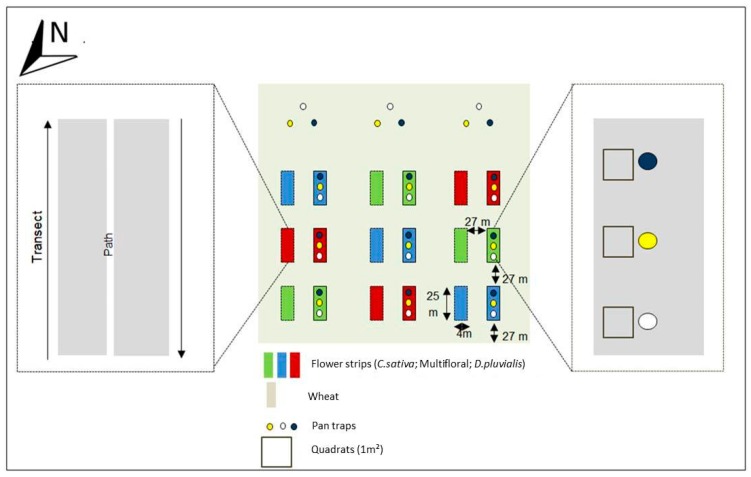
Experimental setup.

Three flower strip treatments were established by sowing a multifloral, and two distinct monofloral, strips (i.e., one with *C. sativa* and one with *D. pluvialis*) in a 12-ha field of the AgricultureIsLife experimental farm of Gembloux Agro-Bio Tech (University of Liege, Belgium) (50°30′52.403″ N; 4°43′51.153″ E). The surrounding landscape was mostly composed of large urbanised areas (52%) and agricultural fields (39%) within a 3 km radius from the field (Figure S1). The multifloral treatment contained 11 floral species that were selected for their melliferous potential: *Daucus carota* (L., 1753) (Apiaceae), *Oenothera biennis* (Linnaeus, 1753) (Onagraceae), *Echium vulgare* (L., 1753) (Boraginaceae), *Coriandrum sativum* (L., 1753) (Apiaceae), *Fagopyrum esculentum* (Moench, 1794) (Polygonaceae), *Glebionis segetum* ((L.) Fourr.,1869) (Asteraceae), *Silene latifolia alba* (Poiret, 1789) (Caryophyllaceae), *Malva moschata* (L., 1753) (Malvaceae), *Geranium pyrenaicum* (Burman, 1753) (Geraniaceae), *Trifolium incarnatum* (L., 1753) (Fabaceae), *Trifolium repens* (L., 1753) (Fabaceae). *T. repens* and *T. incarnatum* were especially chosen for their soil cover properties. To ensure high floral diversity and evenness, the seed mixture was assembled using an equal number of seeds for each floral species (Table S1). Eighteen flower strips (4 m × 25 m) were sown in the field on 27 April 2017, each strip being separated by 27 m of winter wheat, finally constituting an intercropping system. Each floral treatment was repeated three times in a Latin square design and each repetition consisted of two similar adjacent strips (Figure 1). All sampled flower strips were assumed as independent replicates. Winter wheat was sown in November 2016 and no insecticides were used during the experiment.

### 2.2. Pollinator Trapping and Identification

All sampling and identification were limited to bees and hoverflies. These families are the ones participating mainly in the pollination process in an effective and substantial way [5].

Sampling was conducted during a period of three months, from May (early blooms) to July 2017. A standard protocol for pollinator surveys [36] was used: a combination of white, blue and yellow coloured pan traps (Flora^®^, 27 cm diameter and 10 cm depth) were installed every 5 m in the centre of the western strip in each block (Figure 1) every 15 days from 9:00 a.m. to 5:00 p.m. in good weather conditions (i.e., temperature above 15 °C, wind speed below 15 km/h and a clear sky) [37]. To be able to offset the effect of flower strips from the background pollinator community, three lots of pan traps were placed in the wheat field, 40 m away from the flower strips. Pan traps were filled with water and some drops of colourless and odourless detergent (wash liquid ‘Rainett—Ecologique^®^’) to decrease the surface tension of the water. Insects were collected and kept in 70% ethanol. Additionally, floral visitations were assessed through standardised transects conducted from 11:00 a.m. to 12:00 a.m. and from 2:00 p.m. to 3:00 p.m. (i.e., within the range of the wild bee daily peak of activity) [38,39]. Transects were run in each eastern strip of each block (Figure 1). Two walks were undertaken for each floral species with a waiting time of two seconds on every floral unit to observe the visits of pollinators (Figure 1). A floral unit corresponded to one or a set of flowers where the insect can move by walking without needing to fly. When a pollinator landed on a floral unit, it was collected by using a net and kept in a box containing crushed ice. In the laboratory, all collected individuals were preserved in a freezer at −20 °C. The transects were repeated twice, spaced a week apart for each flower species during their flowering time. A total of six days of collection with coloured pan trap traps were made and two net traps for each flower species during flowering, for a total of eight transects (on *C. sativa*, *D. pluvalis*, *C. coriander* and *F. esculentum*). The collected data were encoded separately, depending on the type of flower strip and sampling technique.

The insects were pinned using a pre-established protocol [40]. Insect identification was performed with identification keys [40,41,42,43,44] and with the help of specialists for species checking and specific taxon groups (Halictidae: Alain Pauly; Syrphidae: Frank Van de Meutter; Andrenidae: David Genoud).

### 2.3. Vegetation Surveys

To survey the vegetation development, three quadrats of 1 m × 1 m were placed in each of the western strip of each block (Figure 1) [45]. The number of plants and floral units were counted in each quadrat for every species on 11 July 2017 (i.e., when most of the plants were blooming).

### 2.4. Statistical Analyses

Data analyses were performed with Microsoft Excel 2010 and R software v.3.0.1 [46].

First, the structure of the sampled communities was evaluated with a combination of pan trap and sweep net, and also separately, by considering the abundance of individuals, their species richness and by calculating the following three alpha diversity indexes: Simpson, Shannon and Pielou (‘Vegan’ package [47,48]). The Simpson index calculates the proportion to which two individuals have accumulated in a community of the same species (Simpson, 1949). It takes into account the abundance of each species in a sample and their proportion in the population. The Shannon index (H) is associated with the Simpson index [49]. The proportion of each species is multiplied by its own logarithm. The Shannon index takes better account of important variations of the rarest species [49]. The Pielou (R) index, often complementary to the Shannon index, calculates the distribution of individuals between species or the evenness, regardless of species richness [49]. Because our sample size (*N* = 3 per floral treatment) is too small and normality of our data was not met, a non-parametric test (Kruskal-Wallis; *p*-value < 0.05) was used to assess abundance, species richness and the effects of the alpha diversity indices (i.e., Simpson, Shannon, Pielou) between each treatment (i.e., multifloral, *C. sativa*, *D. pluvialis*) on (i) bees + hoverflies trapped and netted, (ii) bees + hoverflies trapped (iii) bees + hoverflies netted, (iv) bees trapped and netted and (v) hoverflies trapped and netted. These non-parametric tests were followed by post hoc comparisons (Dunn’s test) if necessary, to check for pairwise significant differences. Post-hoc comparisons were computed via the ‘dunn.test’ package [50].

Each local community is supposed to be limited in size with defined species number [51]. Observed species richness from sampling effort (net + coloured pan traps) is dependent on the sample size. Indeed, new species detection expands with the increase of sample size or sampling effort. To check whether the sampling was conducted in a complete manner, sample coverage curves were plotted for: (i) hoverflies and bees together; (ii) bees alone; and (iii) hoverflies alone (‘iNEXT’ package).

Second, the structure of the same sampled communities was evaluated by considering the abundance of individuals and sequence of Hill number [52,53] to compare alpha diversity estimations of the floral treatments (‘iNEXT’ and ‘Vegan’ packages) [47,48]. Indeed, studies proposed a unified framework regarding Hill numbers extended [53] from works based on rarefaction and extrapolation (R/E) sampling curve for species richness and sample completeness [54,55]. Each Hill number corresponds to a diversity order q, which defines species diversity measures as a particular feature: species richness (N = 0), the exponential of the Shannon entropy (N = 1) and the inverse Simpson concentration index (N = 2) [52]. R/E curves were built specifying 100 bootstrap replications on individual-based abundance data to compare the pollinator communities between the floral treatments: (i) hoverflies and bees together, (ii) bees alone and (iii) hoverflies alone.

Third, the structure of the pollinator community in the three treatments was examined through ordination methods using Principal Coordinate Analysis (PCoA) based on Bray-Curtis distance (functions ‘cmdscale,’ ‘ordiplot’ and ‘ordiellipse from the ‘Vegan’ package [47,48]). Data of the pan traps and those from the sweep net were analysed separately. The same analysis was realised for the structure of the pollinator community by floral species using data of the sweep net. The two main components most adequately explaining the variance of the community structures were used to build the PCoA biplots. The community dataframe was standardised using the ‘Hellinger’ method for a one-way analysis of similarities (ANOSIM) also based on Bray-Curtis distance. For every PCoA, ANOSIM was conducted with 9999 permutations to analyse dissimilarity patterns between treatments and flowers.

## 3. Results

### 3.1. Pollinator Diversity in Flower Strips

In total, 1184 pollinator individuals belonging to 61 species were collected with pan traps and the net, of which 18 species were hoverflies (583 individuals) and 43 species were bees (601 individuals). The species accumulation curves, reaching a plateau of saturation, show that the sampling effort was sufficient to collect most of the pollinator diversity of the environment (Figure S2). *Sphaerophoria scripta* (Linnaeus, 1758) (Diptera: Syrphidae) was the most abundant species, followed by *Eristalis tenax* (Linnaeus, 1758) (Diptera: Syrphidae), *Lasioglossum pauxillum* (Schenck, 1853) (Hymenoptera: Halictidae), *Lasioglosssum morio* (Fabricius, 1793) (Hymenoptera: Halictidae) and *Andrena flavipes* (Panzer, 1799) (Hymenoptera: Andrenidae) (Table 1). No rare species were present, except for *Andrena nitidiuscula* (Schenck, 1853) (Hymenoptera: Andrenidae) ranked as minor concern (LC) on the European Red List [56]. Concerning hoverflies, the conservation statuses could not be indicated because no red list at the moment exists for this family.

Simpson, Shannon and Pielou indexes describing alpha diversity showed high diversity for each floral treatment (Table 2) against total species composition of the experimental field. These indexes also exposed that individuals are distributed with several dominant species (Table 1) which reduced community evenness.

The Kruskal-Wallis tests carried out to compare abundance, species richness, Simpson, Shannon and Pielou indexes in the three flower strip treatments showed no significant evidence of a difference between the mean ranks of at least one pair of groups (Table 2). However, the abundance of specimens and Pielou’s evenness index showed a non-significant trend (*p*-value ≤ 0.08) to be distinct, suggesting that it would be different pollinator communities among the floral treatments.

The diversity indexes were also analysed with pan trap and sweep net data separately. No significant difference was found. When bees and hoverflies were analysed separately, there was significant evidence of differences for Simpson and Shannon indexes with hoverfly data (Table 2).

**Figure 2:**
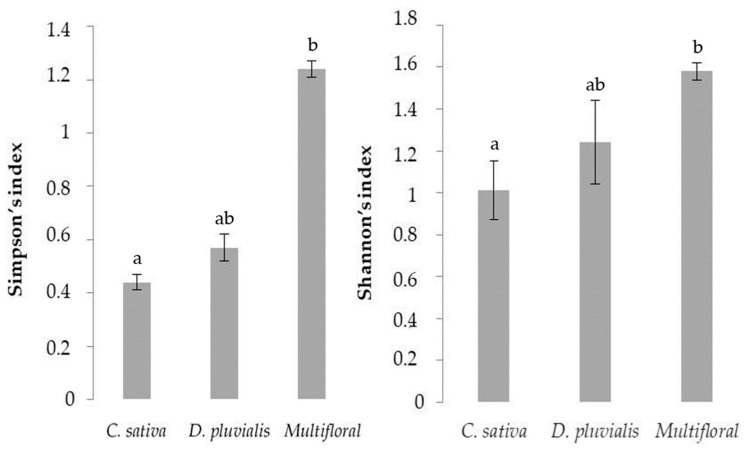
Bar plots of mean values of both Simpson and Shannon indexes for the different treatments. The different letters represent a significant difference calculated from the post-hoc Dunn’s test comparison (*p*-value < 0.05).

Finally, the post-hoc Dunn’s test reveals significant differences between the multifloral treatment and the *C. sativa* treatment for both Simpson (*p*-value = 0.003) and Shannon indexes (*p*-value = 0.005) *(*Figure 2).

**Figure 3:**
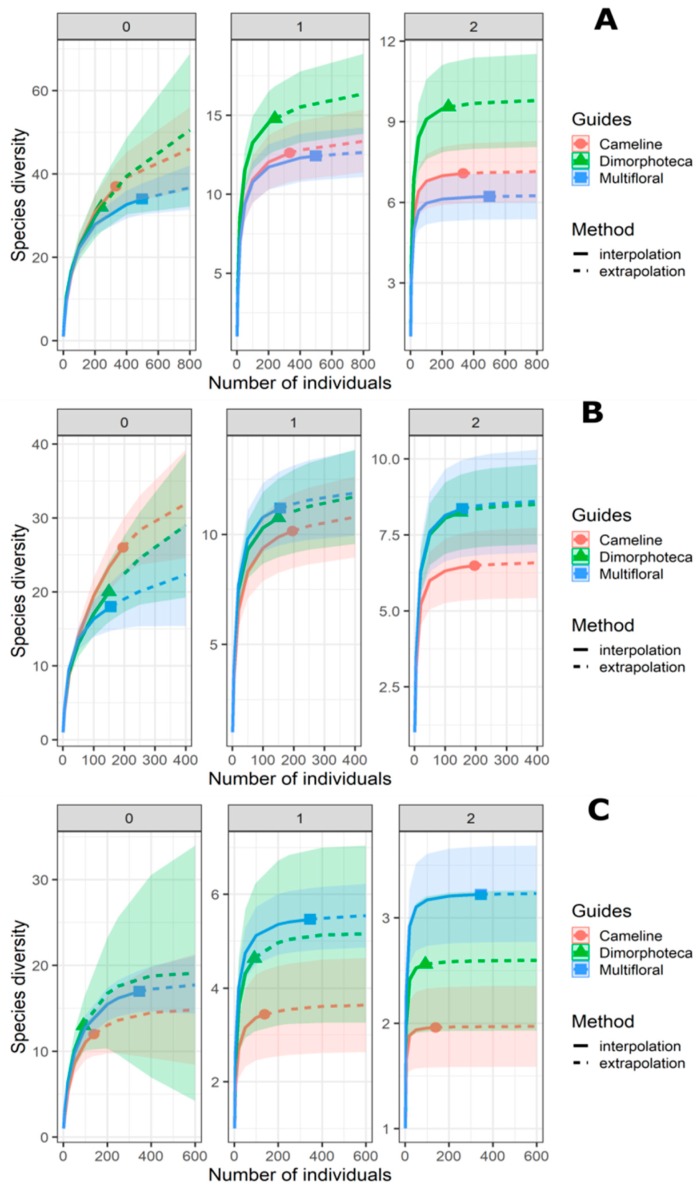
Comparison between pollinator communities from the three floral treatments (denoted by colours and solid dots) by sample-size-based rarefaction (solid lines) and extrapolation (dashed curves) curves based on abundance data of hoverflies and bees together (**A**), bees alone (**B**) and hoverflies alone (**C**). Each panel displays Hill numbers of order N = 0 (left panel), N = 1 (middle panel) and N = 2 (right panel). The 95% confidence intervals (coloured-shaded regions) were obtained by a bootstrap method based on 100 replications.

Rarefaction/extrapolation curves for Hill numbers show that treatments have similar species richness (N = 0) (Table 3, Figure 3). In contrast, there is a significant difference for N = 2 between Dimorphoteca and the other two treatments for hoverflies and bees combined, as suggested by an overlap in the confidence intervals [57]. For both Shannon (N = 1) and Simpson diversities (N = 2), there is a significant difference between multifloral treatment and the other two treatments (Table 3, Figure 3).

**Figure 4:**
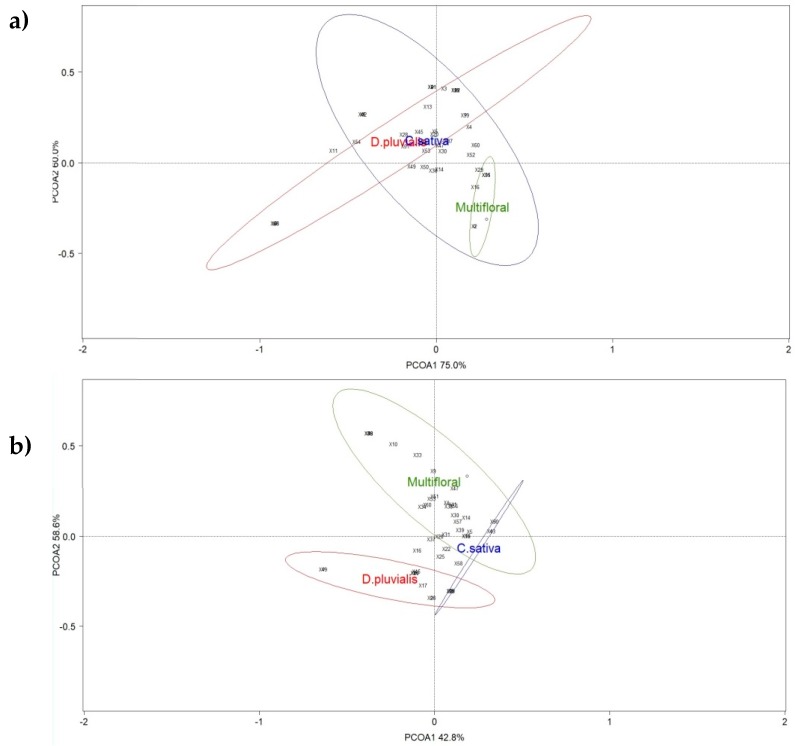
Principal coordinate analysis (PCoA) ordination of the three treatments (red circle: *D. pluvialis*; green circle: Multifloral; blue circle: *C. sativa*) based on the data collected with (**a**) pan traps and (**b**) a net through transects. Ellipses show the 80% confidence interval of the locations grouped by flower strip. Species scores are represented with numbers (Table S2).

ANOSIM show no significant dissimilarities in the pollinator communities in the pan traps (global R = −0.037; *p*-value = 0.606) (Figure 4a). As for the communities captured with the net during transects, the species distribution differed between the three treatments (global R = 0.794; *p*-value = 0.003) (Figure 4b).

### 3.2. The Flower Identity Effect on Pollinator Visitations

**Figure 5:**
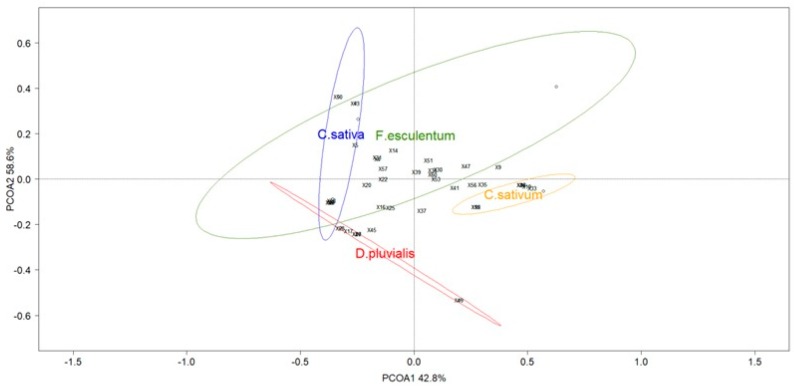
Principal coordinate analysis (PCoA) ordination of the four flower species with data collected with a net (red circle: *D. pluvialis*; yellow circle: *C. sativum*; green circle: *F. esculentum*; blue circle*: C. sativa*). Ellipses show the 80% confidence interval of the locations grouped by flower species. Species scores are represented with numbers (Table S2).

During the transect samplings, coriander *C. sativum* and buckwheat *F. esculentum* were the most abundant species blooming in the multifloral strips (Figure S3). Species richness of netted specimens during transects was composed of ten hoverfly species and 16 bee species. ANOSIM showed differences between the pollinator diversity and abundance of flowers studied (global R = 0.713; *p*-value < 0.001) (Figure 5). Flowers of *D. pluvialis* differed from other flowers. The same is true for *C*. *sativum* and *F. esculentum* flowers. Only *C. sativa* showed a tendency to attract the same pollinator community as *F. esculentum*.

## 4. Discussion

### 4.1. The Biodiversity of Pollinators

1184 individuals belonging to 43 bee species and 18 hoverfly species were collected, representing 11.75% and 5.13% of the national richness in Belgium, respectively [56,58]. These figures are rather low yet considering that land use within a 3 km radius from the field consisted mainly of urbanised areas (52%) and agricultural fields (39%) (Figure S1), such a poor pollinator community is not unexpected [59,60,61]. Indeed, studies have already shown that pollinator species diversity and abundance generally decrease with landscape simplification, leading to a homogenisation of the insect communities [62,63]. The presence of small shrubs, hedges and fragments of woodland on the remaining 8% of the surface area may have provided the necessary resources of nectar and pollen, nesting sites or larval habitat to support a pollinator community, albeit impoverished to some extent [64]. This observation may also explain the low presence of oligolectic bees in our study. Indeed, while polylectic bees are less sensitive to agricultural intensification and the increase of urbanised zones, oligolectic bees (which are less flexible in their range of food resources) are more likely to be affected by agricultural and urban stresses, causing a decline in their population [65].

*Sphaerophoria scripta*, *E. tenax*, *L. pauxillum*, *L. morio* and *A. flavipes* were the most abundant pollinator species. They are all polylectic species common in agricultural landscapes and are recognised as efficient pollinators [43,66]. The composition of the surrounding landscape (i.e., urban areas and agricultural fields) can explain their presence in the field. For example, *L. morio* does not present any particular requirements and nests in anthropogenic areas such as town parks and gardens [43,67]. With regard to hoverfly species, the high abundance of *S. scripta* is consistent with its ecology: it colonises open landscapes with a short turf and patches of bare ground and often frequents pioneer vegetation which makes it a typical species of agricultural environments [68]. The larvae of *S. scripta* are aphid predators amongst others on cereals [69,70]. As for the second most abundant hoverfly species, *E. tenax*, its abundance follows from large-scale long-distance migration in summer [71].

As for less common species, *A. nitidiuscula* were collected on coriander in the floral mixture. So far, some 15 observations of *A. nitidiuscula* are known from Belgium. The only previous observation in the area of Gembloux dates from 1989 (Waarnemingen.be, BDFGM_GX and BDFGM_Mons database) (Figure S4). This species is oligolectic on Apiaceae flowers [72] and inhabits a variety of open habitats [73]. Flower strips sown in agricultural fields seems to be such a habitat that can support fragile (meta)populations of relatively rare pollinator species.

### 4.2. Attractiveness of the Floral Mixtures to Pollinators

We were able to detect significant differences among floral treatments with pan trap and sweep net data aggregated with Hill number analyses. A difference between the multifloral treatment and Dimorphoteca with the N = 2 index has been observed, indicating that the pollinator diversity was higher in the multifloral than in the Dimorphoteca strips. Parallel to the analysis of Hill indices, the indices of Simpson, Shannon and Pielou indicated that the floral strips have housed a fairly large number of species dominated by particular taxa, suggesting that floral strips were attractive to pollinators. Finally, according to the Pielou index, the treatments brought together communities of species whose dominance is equitable, with hypothetically the dominance of certain species.

Moreover, PCoA and ANOSIM show that the floral strips revealed different pollinator communities with net capture while pan trapping did not. This result suggests that both field collection methods are complementary to conduct exhaustive pollinator sampling [74].

Metric analyses using only hoverfly data, however, showed significant differences between the treatments of both Shannon and Simpson indexes and Hill numbers N = 1 and N = 2. These results reveal that multifloral strips make it possible to obtain a greater variety of Syrphidae than when using monofloral strips. Moreover, this result indicates that the pollinator community in monofloral strips tends to be more diverse when dominant pollinator species become more relevant and rare or common species are not favoured. These results can be explained by the various blooms occurring in the flower strips.

The counting of floral units in the quadrats indicates that only two species of the multifloral mixture (i.e., buckwheat and coriander) bloomed in abundance (Figure S3). A first reason for the low germination rate could be the drought wave that occurred in Wallonia in spring 2017 [75] which dried up the soil preventing the germination of many species. A second explanation could be the density of weeds, particularly the Lamb’s quarters *Chenopodium* sp. (Amaranthaceae), which is a nitrophilous species common in conventionally cultivated fields (Figure S3). Nevertheless, some pollinators are attracted by Lamb’s quarters, particularly some hoverflies [24]. This phenomenon recalls that weeds in agricultural landscapes can support ecosystem processes and maintaining their diversity is a crucial issue [76]. These results therefore highlight that the correct establishment of sown wildflower strips and their expected effects on insect biodiversity and the related ecosystem processes is not systematic and depends on environmental (abiotic and biotic) parameters.

### 4.3. The Role of Floral Traits

The pollinator communities on the four flower species that bloomed in abundance were different (Figure 3). Previous studies have demonstrated the importance of floral traits in the attraction of pollinators [77,78,79,80], among them the flower colour and the type of the corolla that determines the nectar and pollen accessibility.

The present flower species were white or yellow. These colours are effective in attracting hoverflies and some bees of the genus *Bombus* (Bray. 1957) [80]. Conversely, the blue flowers, absent in our study, would be more conducive to attracting bees [81]. Floral colours could explain the greater presence of hoverflies, especially on buckwheat and coriander. The positive effect of colour on hoverflies may have been supported by the corolla type of these flower species, qualified as ‘flower with open nectar’ and ‘flower with partly hidden nectar’ after the classification of Müller (1881) [82] in the BIOLFLOR database [83]. Indeed, the corolla type determines the availability of nectar for visitors and species with short corolla depth such as umbel flowers (Apiaceae) (e.g., coriander and some Asteraceae like *D. pluvialis*) or with wide corollas such as buckwheat, are attractive to hoverflies and increase their survivorship [78]. Conversely, nectar in narrow corollas such as that of *C. sativa* is accessible to bees, which could explain the increased abundance of these pollinators in this treatment [84]. These observations can explain the significant differences observed with the ANOSIM results for the PCoA representing the pollinator communities for each flower.

## 5. Conclusions and Perspectives

First, the present study provides an additional list of bees and hoverflies found in a typical agricultural landscape dominated by field crops and urban areas in Wallonia, Belgium. It shows that most of the species collected are generalists in terms of habitats. Moreover, the presence of *A*. *nitidiuscula* enhances the interest of the flower strips by favouring less frequent pollinator species. The study also highlights the abundance of aphidophagous hoverflies, which may benefit farmers by naturally controlling aphids (Hemiptera: Aphididae) that are common agricultural pests in the region [85]. This result supports the need for broadening the scope in order that spatial diversification of agro-ecosystems addresses multiple issues simultaneously [28].

Second, the study did not generally reveal significant differences in terms of abundance and diversity of pollinators in the different treatments (i.e., monospecific vs. multifloral strips). Only hoverflies were more diversified (Shannon’s and Simpson’s diversity) in the multifloral mixture. A reason may be that few species in the multifloral mixture actually bloomed. In addition, this study was conducted on a single experimental site, which makes it impossible to compare the results between different experimental fields that could have shown significant differences in terms of pollinators and species blooms. Further studies are thus required to draw a clearer conclusion on whether multispecies wildflower mixtures or monofloral crops benefit pollinators the best. In particular, flower phenology remains a key element of the effectiveness of flowering strips. Hence, further research should assess the effect of blooming time on pollinator species emerging early in the season as well as on those requiring food resources late in the season. Moreover, it would be useful to evaluate whether an earlier or later sowing of *C. sativa* and *D. pluvialis* would allow their flowering to be spread out over a longer period.

Third, the significant difference of pollinator communities observed on each flower species reinforces the interest of identifying the floral traits benefiting visiting insects to improve floral blends.

Being conducted in a single year, this work could be completed in the future by exploring the evolution of the obtained results on a longer term. Finally, the economic benefits provided by the cultivation of *C. sativa* and *D. pluvialis* could be compared with the monetary compensation provided to farmers by the AECM for multifloral mixtures. Proving the economic profitability of sowing flower strips could encourage farmers to diversify their agricultural systems as well as their incomes.

## Figures and Tables

**Table 1:** Abundance of all bee and hoverfly species collected with pan traps and during transects in each treatment. The endangered status from the European red list of bees [56] for each bee species is indicated (LC: Minor concern; DD: insufficient data). To our knowledge, no endangered status information is available for hoverflies. Foraging traits are also pointed out (P: Polylectic; O: oligolectic with the family of flower; C: cuckoo bees or kleptoparasites).

Species	Status	Foraging	Multifloral	*C. sativa*	*D. pluvialis*	Control	Total	% Total
*Anthophila*								
*Andrenidae*								
*Andrena carantonica*	*D.D*	P	0	0	0	1	1	0.08
*Andrena chrysosceles*	*D.D*	P	1	0	0	0	1	0.08
*Andrena cineraria*	*L.C*	P	0	1	1	0	2	0.17
*Andrena dorsata*	*D.D*	P	5	1	3	0	9	0.76
*Andrena flavipes*	*L.C*	P	23	47	14	2	86	7.29
*Andrena gravida*	*D.D*	P	0	2	0	0	2	0.17
*Andrena haemorrhoa*	*L.C*	P	0	0	1	0	1	0.08
*Andrena humilis*	*D.D*	O Asteraceae	0	0	0	1	1	0.08
*Andrena minutula*	*D.D*	P	10	1	0	1	12	1.02
*Andrena minutuloides*	*D.D*	P	4	0	0	0	4	0.34
*Andrena nigroaenea*	*L.C*	P	0	2	1	0	3	0.25
*Andrena nitida*	*L.C*	P	0	1	2	0	3	0.25
*Andrena nitidiuscula*	*L.C*	O Apiaceae	1	0	0	0	1	0.08
*Apidae*								
*Apis mellifera*	*L.C*	P	6	5	3	5	19	1.61
*Bombus hypnorum*	*L.C*	P	0	0	0	1	1	0.08
*Bombus lapidarius*	*L.C*	P	7	5	14	2	28	2.37
*Bombus lucorum*	*L.C*	P	0	0	4	0	4	0.34
*Bombus pascuorum*	*L.C*	P	0	1	0	0	1	0.08
*Bombus pratorum*	*L.C*	P	0	0	0	1	1	0.08
*Bombus sylvestris*	*L.C*	P	0	0	1	0	1	0.08
*Bombus terrestris*	*L.C*	P	16	9	12	3	40	3.39
*Bombus vestalis*	*L.C*	C	0	1	0	0	1	0.08
*Nomada fabriciana*		C	0	0	0	1	1	0.08
*Colletidae*							
*Hylaeus* sp.	*L.C*	-	1	1	0	0	2	0.17
*Crabronidae*								
*Lindenius* sp.	*L.C*	-	2	0	0	0	2	0.17
*Oxybelus* sp.	*L.C*	-	0	0	0	1	1	0.08
*Halictidae*								
*Halictus maculatus*	*L.C*	P	0	1	0	0	1	0.08
*Halictus rubicundus*	*L.C*	P	0	2	0	0	2	0.17
*Halictus scabiosae*	*L.C*	O Asteraceae	0	0	0	1	1	0.08
*Lasioglossum calceatum*	*L.C*	P	4	7	25	1	37	3.14
*Lasioglossum fulvicorne*	*L.C*	P	2	2	0	3	7	0.59
*Lasioglossum laticeps*	*L.C*	P	6	5	0	3	14	1.19
*Lasioglossum leucozonium*	*L.C*	P	0	0	0	1	1	0.08
*Lasioglossum malachurum*	*L.C*	P	10	12	13	4	39	3.31
*Lasioglossum minutissimum*	*L.C*	P	0	3	0	0	3	0.25
*Lasioglossum morio*	*L.C*	P	19	36	18	41	114	9.66
*Lasioglossum nitidulum*	*L.C*	P	0	0	1	0	1	0.08
*Lasioglossum nitidiusculum*	*L.C*	P	0	1	1	0	2	0.17
*Lasioglossum pauxillum*	*L.C*	P	37	45	32	24	138	11.69
*Lasioglossum villosulum*	*L.C*	P	0	2	1	0	3	0.25
*Seladonia tumulorum*	*L.C*	P	1	2	1	0	4	0.34
*Sphecodes ephippius*	*L.C*	C	1	0	0	0	1	0.08
*Sphecodes monilicornis*	*L.C*	C	2	0	0	0	2	0.17
*Syrphidae*								
*Episyrphus balteatus*	-	P	16	4	6	0	26	2.20
*Eristalis arbustorum*	-	P	11	0	0	0	11	0.93
*Eristalis similis*	-	P	0	0	0	1	1	0.08
*Eristalis tenax*	-	P	70	12	56	3	141	11.95
*Eumerus strigatus*	-	P	7	4	0	2	13	1.10
*Eupeodes corolla*	-	P	7	3	2	0	12	1.02
*Eupeodes latifasciatus*	-	P	0	0	1	0	1	0.08
*Eupeodes luniger*	-	P	7	0	0	0	7	0.59
*Melanostoma mellinum*	-	P	3	6	7	0	16	1.36
*Platycheirus clypeatus*	-	P	2	1	4	0	7	0.59
*Scaeva pyrastri*	-	P	9	0	3	0	12	1.02
*Scaeva selenitica*	-	P	0	0	1	0	1	0.08
*Sphaerophoria rueppelli*	-	P	1	3	1	0	5	0.42
*Sphaerophoria scripta*	-	P	176	98	7	8	289	24.49
*Sphaerophoria taeniata*	-	P	3	3	0	0	6	0.51
*Syritta pipiens*	-	P	25	3	0	0	28	2.37
*Syrphus ribesii*	-	P	3	0	1	0	4	0.34
*Syrphus vitripennis*	-	P	1	1	1	0	3	0.25

**Table 2:** Mean abundance and species richness of pollinator community, diversity (Simpson, Shannon and Pielou) depending on the type of collection and pollinator family in each treatment (±standard deviation), the degree of freedom (df), Kruskal-Wallis, χ^2^-value and significant differences (*: *p*-value < 0.05).

Data		*C. sativa*	*D. pluvialis*	Multifloral	df	χ^2^	*p*-Value
Pan traps *net* hoverflies*bees	Abundance	111.00 ± 31.43	79.30 ± 23.46	165.67 ± 54.99	2	5.07	0.08
	Species richness	22.67 ± 3.79	19.33 ± 2.08	26.67 ± 1.16	2	4.47	0.12
	Simpson’s Diversity	0.82 ± 0.06	0.88 ± 0.03	0.83 ± 0.04	2	3.29	0.19
	Shannon’s Diversity	2.31 ± 0.23	2.47 ± 0.15	2.41 ± 0.16	2	1.16	0.67
	Pielou’s evenness	0.74 ± 0.04	0.84 ± 0.04	0.74 ± 0.06	2	5.42	0.07
Pan traps* hoverflies*bees	Abundance	34 ± 24.75	18.3 ± 5.85	17 ± 1.00	2	0.97	0.61
	Species richness	9.33 ± 2.88	9.66 ± 1.52	8.66 ± 1.52	2	0.85	0.65
	Simpson’s Diversity	0.76 ± 0.08	0.81 ± 0.08	0.81 ± 0.08	2	1.15	0.56
	Shannon’s Diversity	1.75 ± 0.35	1.98 ± 0.37	1.94 ± 0.31	2	0.62	0.73
	Pielou’s evenness	0.8 ± 0.10	0.87 ± 0.10	0.9 ± 0.07	2	2.22	0.32
Net *hoverflies*bees	Abundance	59 ± 10.58	45.33 ± 27.64	138.33 ± 59.80	2	5.60	0.06
	Species richness	14 ± 2.00	11.6 ± 3.78	21.66 ± 1.52	2	5.80	0.06
	Simpson’s Diversity	0.71 ± 0.03	0.78 ± 0.02	0.78 ± 0.02	2	5.42	0.06
	Shannon’s Diversity	1.81 ± 0.10	1.93 ± 0.11	2.18 ± 0.18	2	5.06	0.07
	Pielou’s evenness	0.68 ± 0.02	0.80 ± 0.06	0.70 ± 0.05	2	4.62	0.09
Pan taps*net *bees	Abundance	48.30 ± 33.60	44.00 ± 7.00	42 ± 1.00	2	2.98	0.22
	Species richness	12.00 ± 3.00	10.00 ± 0.00	13 ± 1.00	2	3.08	0.21
	Simpson’s Diversity	0.78 ± 0.01	0.83 ± 0.01	0.84 ± 0.01	2	5.95	0.06
	Shannon’s Diversity	1.88 ± 0.16	2.00 ± 0.03	2.18 ± 0.08	2	5.60	0.06
	Pielou’s evenness	0.76 ± 0.08	0.86 ± 0.01	0.84 ± 0.02	2	3.20	0.20
Pan traps*net *hoverflies	Abundance	44.26 ± 11.67	29.66 ± 21.36	113.33 ± 6.18	2	5.95	0.05
	Species richness	7.00 ± 1.73	6.66 ± 2.88	12.33 ± 1.52	2	5.65	0.05
	Simpson’s Diversity	0.44 ± 0.03	0.57 ± 0.05	1.24 ± 0.03	2	7.20	0.02 *
	Shannon’s Diversity	1.01 ± 0.14	1.24 ± 0.20	1.58 ± 0.04	2	6.48	0.03 *
	Pielou’s evenness	0.53 ± 0.02	0.68 ± 0.06	0.63 ± 0.03	2	5.95	0.05

**Table 3:** Hill diversity indices of each treatment based on abundance data of hoverflies and bees together, bees alone and hoverflies alone where N0 = species richness; N1 = evenness; N2 = diversity weighted by relative abundance.

		Bees + Hoverflies	Bees	Hoverflies
**Camelina**	N0	914	713	832
	N1	1261	339	981
	N2	839	225	76
**Dimorphoteca**	N0	2307	1006	1306
	N1	1477	188	1049
	N2	1193	351	992
**Multifloral**	N0	646	527	371
	N1	1242	563	1067
	N2	727	375	1033

## Supplementary Materials

The following are available online at http://www.mdpi.com/2075-4450/9/3/114/s1, Table S1: Floral mixtures of the three treatments, Table S2: Species scores of the PCOA, Figure S1: Mapping of the landscape around the experimental field on a radius of 3 km, Figure S2: Species accumulation curves based on abundance data of hoverflies and bees together (A), bees alone (B) and hoverflies alone (C), Figure S3: Number of floral units per floral species in the quadrats of multifloral flower strips, Figure S4: Mapping of the Andrena nitidiuscula distribution in Belgium since 1929 (Source: Rasmont (2017); Atlas Hymenoptera).
