# Supplementary material for: Flower Strips in Wheat Intercropping System: Effect on Pollinator Abundance and Diversity in Belgium
Source: Insects. 2018 Sep 4;9(3):114. doi: 10.3390/insects9030114 (PMC6164983; doi:10.3390/insects9030114)
Supplement: Supplementary file 1 [file insects-09-00114-s001.pdf]

Supplementary Materials: The following are available online at www.mdpi.com/xxx/s1, Table S1: Floral mixtures of the three treatments, Table S2: Species scores of the PCOA, Figure S1: Mapping of the landscape around the experimental field on a radius of 3 km, Figure S2: Species accumulation curves based on abundance data of hoverflies and bees together (A), bees alone (B) and hoverflies alone (C), Figure S3: Number of floral units per floral species in the quadrats of multifloral flower strips, Figure S4: Mapping of the Andrena nitidiuscula distribution in Belgium since 1929 (Source: Rasmont (2017); Atlas Hymenoptera).

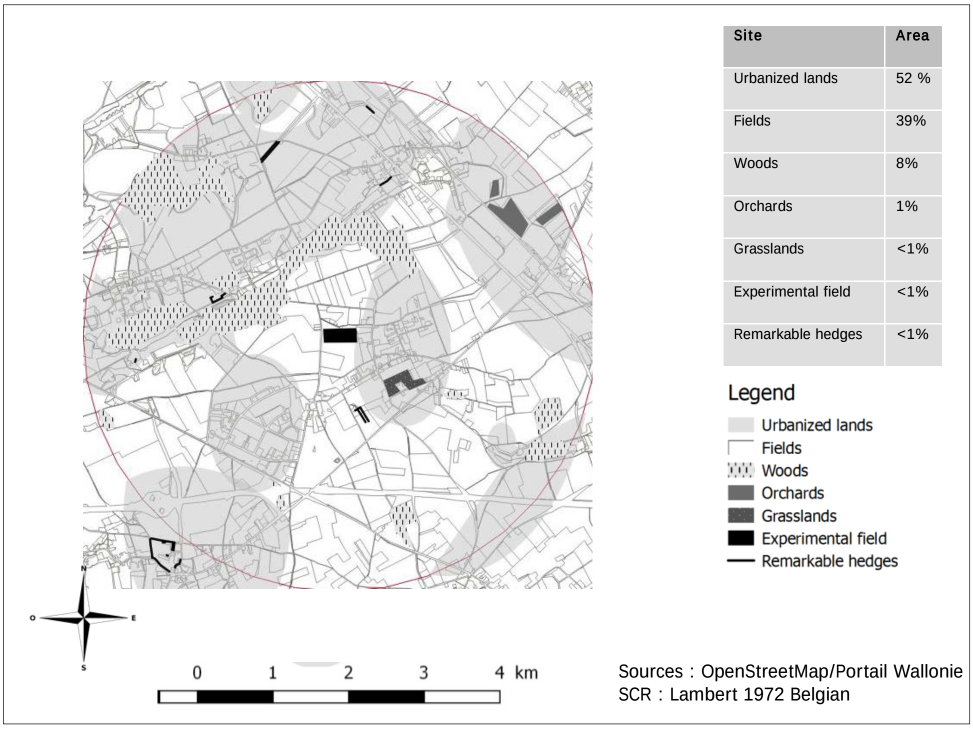

Site	Area
Urbanized lands	52 %
Fields	39%
Woods	8%
Orchards	1%
Grasslands	<1%
Experimental field	<1%
Remarkable hedges	<1%

Figure S1 Mapping of the landscape around the experimental field on a radius of 3 km

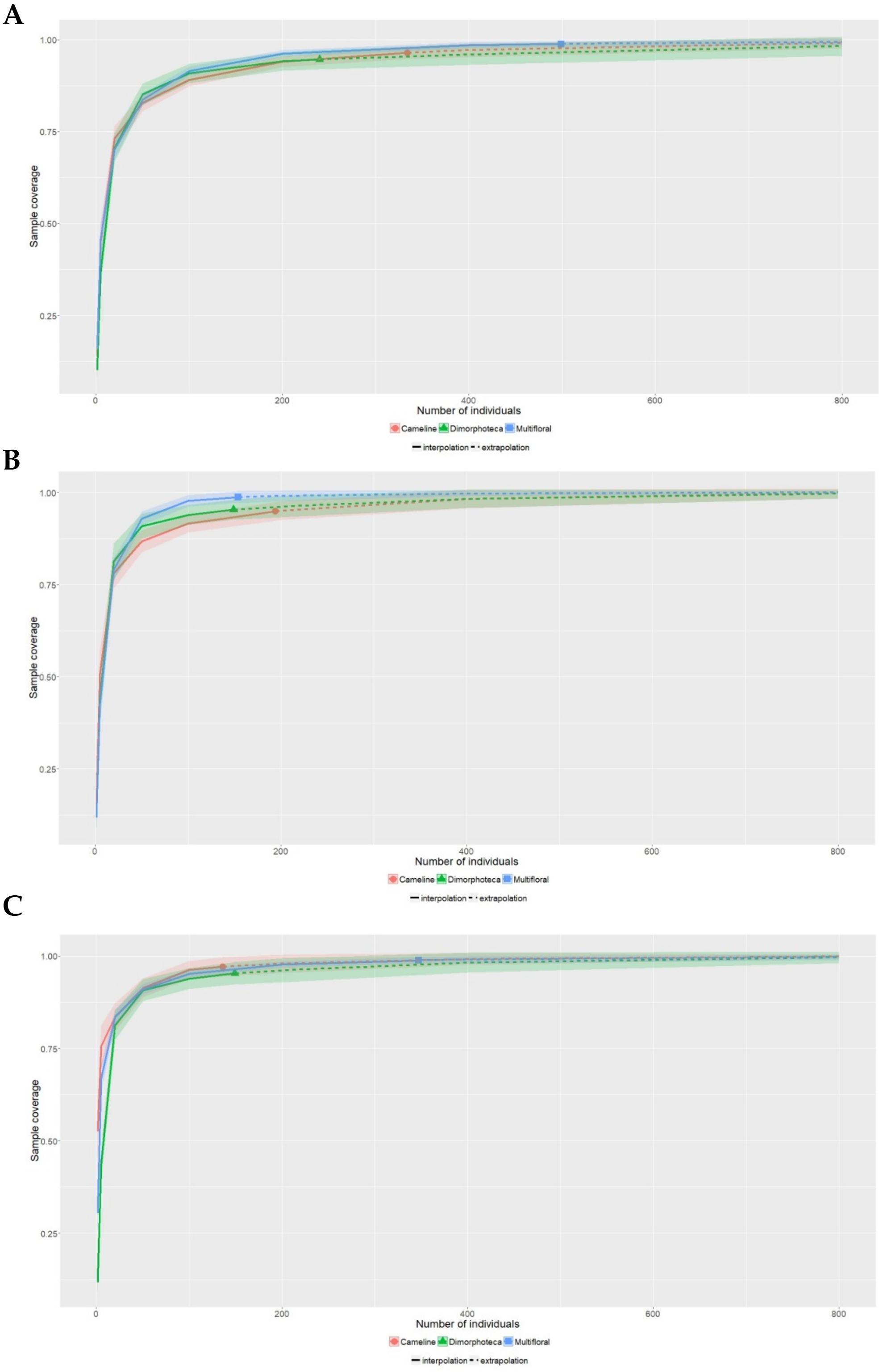

Figure S2 Species accumulation curves based on abundance data of hoverflies and bees together (A), bees alone (B) and hoverflies alone (C)

Table S 1 Floral mixtures of the three treatments

**Treatment 1**	**Kind Species**	**Phenology**	**Weight (g)/ha**	**g/16m²**
**Floral mixture**				
Wild carrot	*Daucus carota*	Biennial	175	0,28
Evening primrose	*Oenothera biennis*	Biennial	70	0,112
Viperine	*Echium vulgare*	Biennial	507,5	0,812
Coriander	*Coriandrum sativum*	Annual	1400	2,24
Buckwheat	*Fagopyrum esculentum*	Annual	1750	2,8
Chrysanthemum of the harvest	*Glebionis segetum*	Annual	157,5	0,252
Whit mate	*Silene latifolia alba*	Sustainable	140	0,224
Mallow	*Malva moschata*	Sustainable	350	0,56
Pyrenees Geranium	*Geranium pyrenaicum*	Sustainable	350	0,56
		**Total**	**4900**	**7,84**
**Background cover**				
Crimson clover	*Trifolium incarnatum*		100	0,16
White clover	*Trifolium repens*		100	0,16
		**Total**	**5000**	**8**
**Treatment 2**				
Dimorphoteca	*Dimorphoteca pluvialis*		5000	8
**Treatment 3**				
Camelina	*Camelina sativa*		5000	8

Table S2 Species scores of the PCOA

1	*Andrena carantonica*	32	*Sphecodes ephippius*
2	*Andrena chrysosceles*	33	*Sphecodes monilicornis*
3	*Andrena cineraria*	34	*Episyrphus balteatus*
4	*Andrena dorsata*	35	*Eristalis arbustorum*
5	*Andrena flavipes*	36	*Eristalis similis*
6	*Andrena gravida*	37	*Eristalis tenax*
7	*Andrena haemorrhoa*	38	*Eumerus strigatus*
8	*Andrena humilis*	39	*Eupeodes corolla*
9	*Andrena minutula*	40	*Eupeodes latifasciatus*
10	*Andrena minutuloides*	41	*Eupeodes luniger*
11	*Andrena nigroaenea*	42	*Halictus maculatus*
12	*Andrena nigroena*	43	*Halictus rubicundus*
13	*Andrena nitida*	44	*Halictus scabiosae*
14	*Apis mellifera*	45	*Lasioglossum calceatum*
15	*Bombus hypnorum*	46	*Lasioglossum fulvicorne*
16	*Bombus lapidarius*	47	*Lasioglossum laticeps*
17	*Bombus lucorum*	48	*Lasioglossum leucozonium*
18	*Bombus pascuorum*	49	*Lasioglossum malachurum*
19	*Bombus pratorum*	50	*Lasioglossum minutissimum*
20	*Bombus terrestris*	51	*Lasioglossum morio*
21	*Bombus vestalis*	52	*Lasioglossum nitidiusculum*
22	*Melanostoma mellinum*	53	*Lasioglossum pauxillum*
23	*Nomada fabriciana*	54	*Lasioglossum villosulum*
24	*Oxybelus sp*	55	*Lindenius sp*
25	*Platycheirus clypeatus*	56	*Syritta pipiens*
26	*Bombus sylvestris*	57	*Syrphus ribesii*
27	*Scaeva selenitica*	58	*Syrphus vitripennis*
28	*Seladonia tumulorum*	59	*Lasioglossum nitidulum*
29	*Sphaerophoria rueppelli*	60	*Scaeva pyrastri*
30	*Sphaerophoria scripta*	61	*Hyaleus sp*
31	*Sphaerophoria taeniata*		

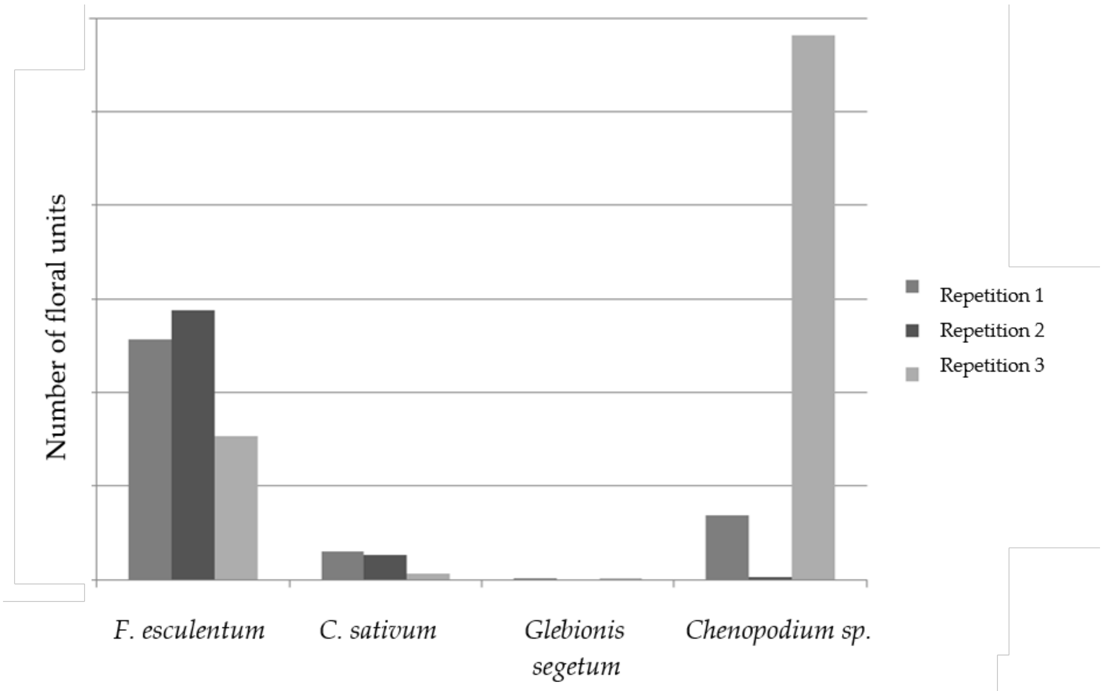

Figure S3 Number of floral units per floral species in the quadrats of multifloral flower strips

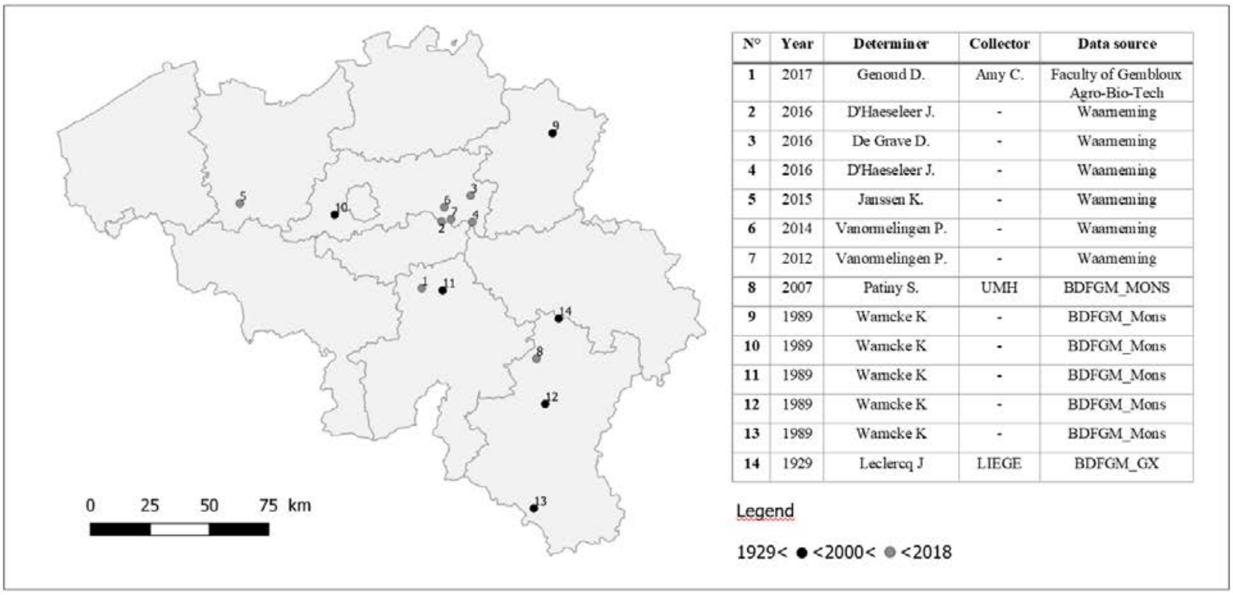

N°	Year	Determiner	Collector	Data source
1	2017	Genoud D.	Amy C.	Faculty of Gembloux Agro-Bio-Tech
2	2016	D'Haeseleer J.	-	Waameming
3	2016	De Grave D.	-	Waameming
4	2016	D'Haeseleer J.	-	Waameming
5	2015	Janssen K.	-	Waameming
6	2014	Vanormelingen P.	-	Waameming
7	2012	Vanormelingen P.	-	Waameming
8	2007	Patiny S.	UMH	BDFGM_MONS
9	1989	Wamcke K	-	BDFGM_Mons
10	1989	Wamcke K	-	BDFGM_Mons
11	1989	Wamcke K	-	BDFGM_Mons
12	1989	Wamcke K	-	BDFGM_Mons
13	1989	Wamcke K	-	BDFGM_Mons
14	1929	Leclercq J	LIEGE	BDFGM_GX

Figure S4 Mapping of the *Andrena nitidiuscula* repartition in Belgium since 1929 (Source: Rasmont (2017); Atlas hymenoptera)
